# Correction: Transplantation of *HGF* gene-engineered skeletal myoblasts improve infarction recovery in a rat myocardial ischemia model

**DOI:** 10.1371/journal.pone.0196413

**Published:** 2018-04-19

**Authors:** Shu-Ling Rong, Xiao-Lin Wang, Cui-Ying Zhang, Zhuo-Hui Song, Lu-Hua Cui, Xiao-Feng He, Xu-Jiong Li, Hui-Jin Du, Bao Li

A portion of the caption for Fig 5 is incorrectly duplicated within the Fig 6 caption. Additionally, the *, ^#^, and † symbols are incorrectly assigned within the captions of Figs 5, 6, 7 and 8. Please see the corrected captions here.

**Fig 5:**
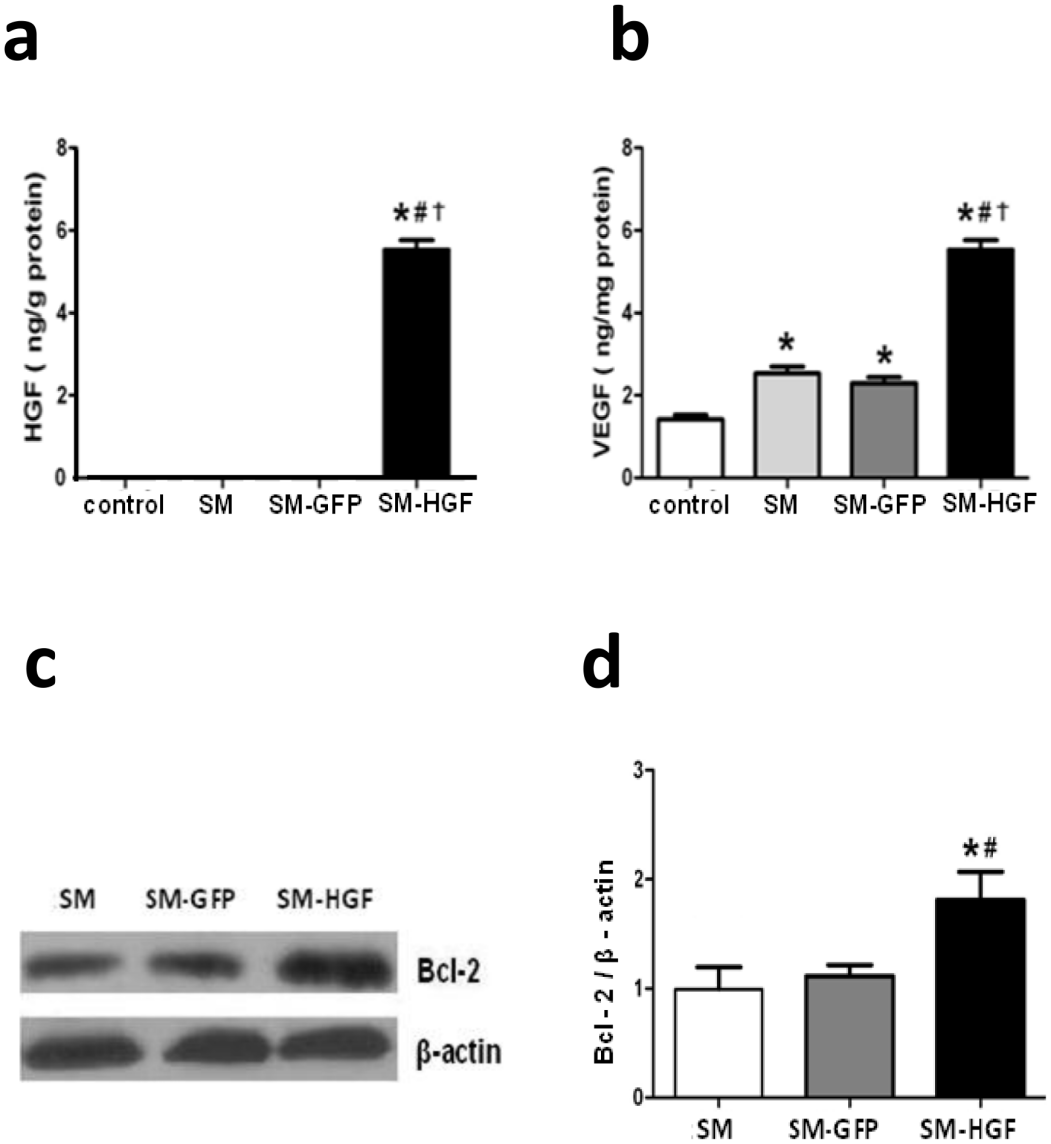
Implantation of HGF-modified skeletal myoblasts increases the HGF and VEGF expression in the myocardium. The Ad-GFP- or Ad-HGF- transduced myoblasts were implanted into the myocardium in an MI model for 7 days. The protein levels of HGF (a) and VEGF (b) in the transplanted area were determined by ELISA. (c) Western blot was performed for Bcl-2 expression in SM transduced with Ad-HGF, vector control, and cell control group. (d) The ratio of Bcl-2 to β-actin in the transplanted area was evaluated by Western blot (Fig 5a and Fig 5b: n = 6, P<0.05, *P<0.05 *vs*. control group; ^#^P<0.05 *vs*. SM group; †P<0.05 *vs*. SM-GFP group. Fig 5d: n = 6, P<0.05, *P<0.05 *vs*. SM group; ^#^P<0.05 *vs*. SM-GFP group).

**Fig 6:**
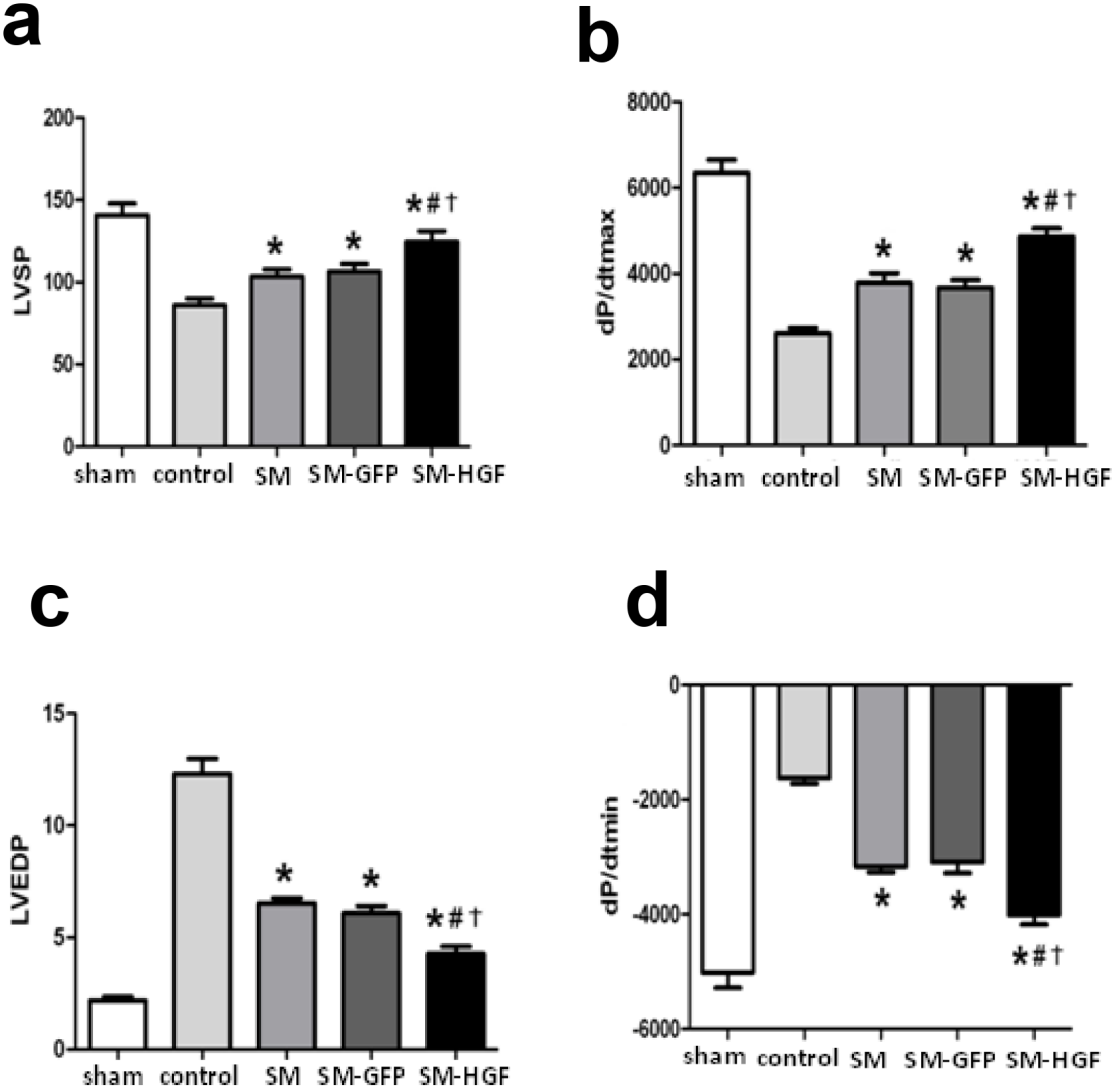
Implantation of HGF-modified skeletal myoblasts improves the heart function. The Ad-GFP or Ad-HGF transduced myoblasts were implanted into the myocardium in an MI model for 28 days. The cardiac functional parameters including LVSP (a) and +dp/dt_max_ (b), LVEDP (c), and dp/dt_min_ (d) were detected (n = 6, P<0.05, *P<0.05 *vs*. control group; ^#^P<0.05 *vs*. SM group; †P<0.05 *vs*. SM-GFP group).

**Fig 7:**
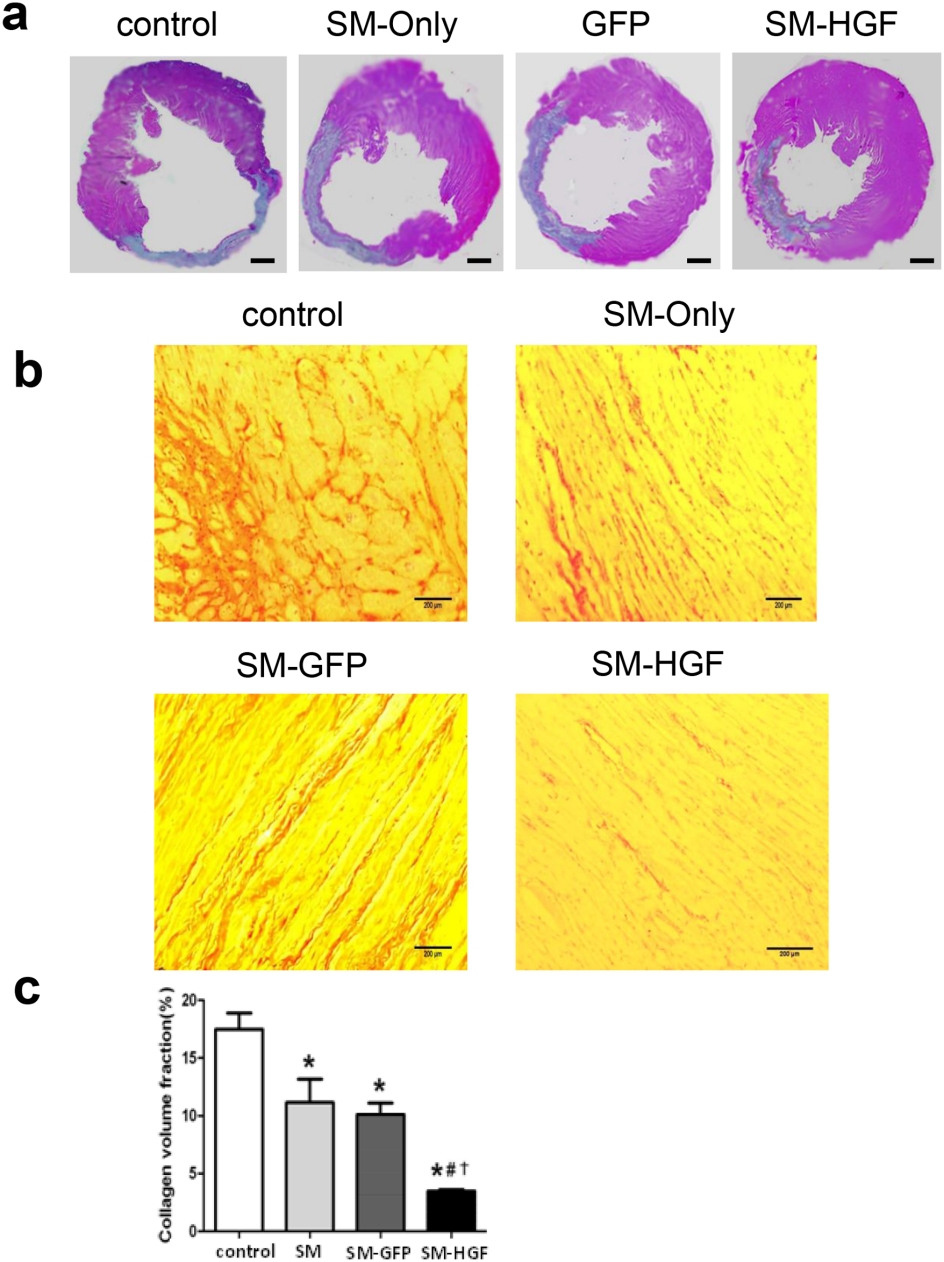
Implantation of HGF-modified skeletal myoblasts reduces infarct area and reduces collagen deposition. The Ad-GFP- or Ad-HGF-transduced myoblasts were implanted into the myocardium in an MI model for 28 days. (a) Representative images of histological sections in left ventricular stained with HE. Scale bars, 1 mm. (b) Images show that Sirius Red stained the cardiac sections from SM-GFP, SM only, SM-HGF group, and sham group, respectively. (c) The collagen volume fraction of SM-HGF group compared to the control group and SM group (n = 6, P<0.05, *P<0.05 *vs*. control group; ^#^P<0.05 *vs*. SM group; †P<0.05 *vs*. SM-GFP group).

**Fig 8:**
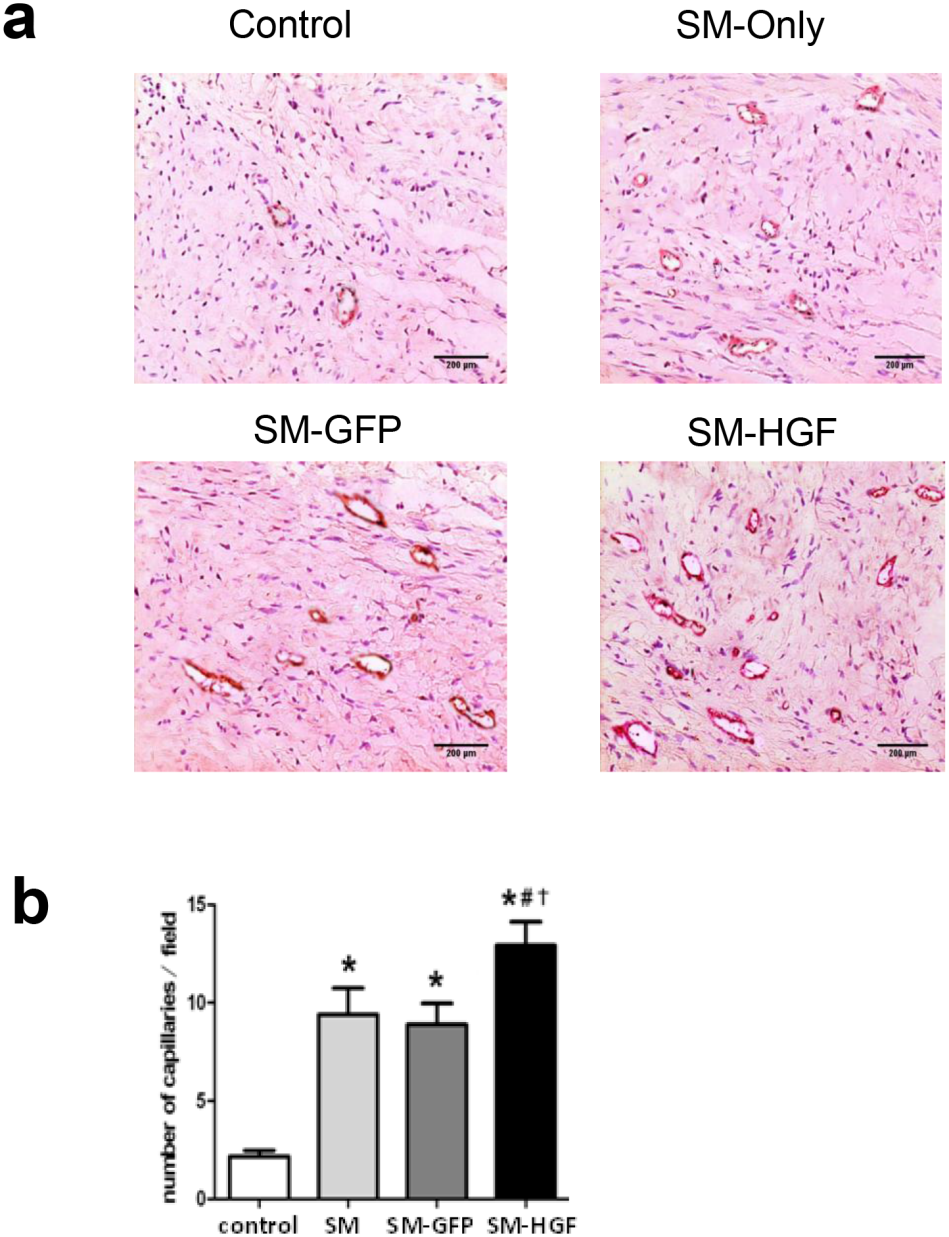
Implantation of HGF-modified skeletal myoblasts increase the vessel density. The Ad-GFP- or Ad-HGF-transduced myoblasts were implanted into the myocardium in an MI model for 28 days. (a) Sections were stained with antibodies against Factor VIII to facilitate the counting of vessels. The representative images of the capillary density in transplanted area were shown. (b) The numbers of vascular densities of groups indicated (n = 6, P<0.05, *P<0.05 *vs*. control group; ^#^P<0.05 *vs*. SM group; †P<0.05 *vs*. SM-GFP group).

## References

[1] Rong S-L, Wang X-L, Zhang C-Y, Song Z-H, Cui L-H, He X-F, et al (2017) Transplantation of *HGF* gene-engineered skeletal myoblasts improve infarction recovery in a rat myocardial ischemia model. PLoS ONE 12(5): e0175807 https://doi.org/10.1371/journal.pone.0175807 28459804 10.1371/journal.pone.0175807 PMC5411067

